# Recent Findings on Therapeutic Cancer Vaccines: An Updated Review

**DOI:** 10.3390/biom14040503

**Published:** 2024-04-21

**Authors:** Sara Sheikhlary, David Humberto Lopez, Sophia Moghimi, Bo Sun

**Affiliations:** 1 Department of Biomedical Engineering, College of Engineering, The University of Arizona, Tucson, AZ 85721, USA; 2 Department of Pharmacology and Toxicology, College of Pharmacy, The University of Arizona, Tucson, AZ 85721, USA; davidlopez3@arizona.edu (D.H.L.); sophiam2@arizona.edu (S.M.)

**Keywords:** cancer vaccines, immunotherapy, cold plasma, stem cells

## Abstract

Cancer remains one of the global leading causes of death and various vaccines have been developed over the years against it, including cell-based, nucleic acid-based, and viral-based cancer vaccines. Although many vaccines have been effective in in vivo and clinical studies and some have been FDA-approved, there are major limitations to overcome: (1) developing one universal vaccine for a specific cancer is difficult, as tumors with different antigens are different for different individuals, (2) the tumor antigens may be similar to the body’s own antigens, and (3) there is the possibility of cancer recurrence. Therefore, developing personalized cancer vaccines with the ability to distinguish between the tumor and the body’s antigens is indispensable. This paper provides a comprehensive review of different types of cancer vaccines and highlights important factors necessary for developing efficient cancer vaccines. Moreover, the application of other technologies in cancer therapy is discussed. Finally, several insights and conclusions are presented, such as the possibility of using cold plasma and cancer stem cells in developing future cancer vaccines, to tackle the major limitations in the cancer vaccine developmental process.

## 1. Introduction

Vaccines have been used to protect human health against infectious diseases since their first discovery in the late 1700s [1]. The recent success of vaccines against the coronavirus disease is encouraging researchers to extend the underlying concepts to treat cancers [2,3]. Active immunotherapy or vaccination is one of the important aspects of efficient tumor eradication by therapeutic cancer vaccines that can be stimulated and enhanced in two major ways: (1) using nonspecific proinflammatory molecules and adjuvants to improve the antitumor immune response already present in the body or (2) provoking a new immune response against specific tumor antigens in the host [4]. The desired tumor antigens and adjuvants are usually delivered together to stimulate adaptive immune systems, aiming to accomplish the optimal activation of dendritic cells (DCs) and durable responses from effector T cells [5]. Innate immune cells, such as natural killer (NK) cells and phagocytes, also play essential roles in tumor recognition and inhibition [6]. However, the immunosuppressive tumor microenvironment (TME) is one of the key obstacles to tumor-infiltrating immune cells and immunotherapies. The combination of therapeutic cancer vaccines and immune checkpoint inhibitors has become the emerging approach to enhance patients’ response rates and survival [7]. The efficacy of cancer vaccines is still under scrutiny in numerous clinical trials [8]. In this review, we explore the mechanism of the cancer immune cycle in the TME and analyze the effectiveness and limitations of major cancer vaccine platforms. Further, we provide new insights for forthcoming cancer vaccines to be more efficient.

## 2. Tumor Microenvironment and Cancer Vaccine Mechanisms

The TME contains a plethora of immune cells, such as monocytes, macrophages, natural killer cells (NKs), dendritic cells (DCs), lymphocyte B cells, and lymphocyte T cells (CD4+ and CD8+) that play key role in the antigen-presentation process and cancer immune cycle that can lead to tumor progression; therefore, targeting the TME and its components is considered a major mechanism for effective cancer vaccines [5,9,10,11]. Macrophages, B cells, and DCs in the TME are some examples of the cells called antigen-presenting cells (APCs). These cells promote antigen-specific immune cell interaction and activation (called priming process) by taking up the antigens originating either from vaccine injections through various administration routes (subcutaneous, intradermal, or intramuscular) or from dead cancer cells. The APCs then make the antigens present on the major histocompatibility complex (MHC) class I or II [12,13,14] (in humans, the human leukocyte antigen (HLA) is the MHC system [15]). This is followed by APC migrations from the TME to the lymph nodes to activate the effector T cells (CD4+ or CD8+) [16,17,18]. Lymph nodes are one of the secondary lymphoid organs (SLOs) that provide a three-dimensional structure for immune cells and enhance the interactions between antigen-loaded APCs and effector T cells to activate T cells and produce an effective immune response. Within the lymph nodes, the mature APCs can activate the effector T cells by presenting the MHC-antigen complexes to the effector T cells. This is followed by the infiltration of the activated effector T cells into the TME, where the T cells can recognize the targeted cancer cells and kill them [19,20,21]. Primary (quiescent) B cell follicles in SLOs (called follicular B cells) become activated upon antigen binding to the primary follicles; following activation, primary follicular B cells turn into the secondary follicles, containing a central germinal center (GC) full of B cell blasts, and with antibody maturation, these B cell blasts undergo several phases and processes pertinent to antibody maturation. These steps lead to the differentiation of lymphocytes into effector T (Teff) cells and B memory cells and in this way, their migrations into the TME is facilitated, leading to the eradication of the tumor cells [5,22,23,24,25,26,27,28,29,30]. However, based on studies on the TME, the generation of antitumor defenses occur not only in SLOs, but also directly within SLO-like aggregations called tertiary lymphoid structures (TLS) [31] that develop in the TME through cytokine accumulations, including CXCL13, RANKL, and interleukin (IL)-7. These structures interact with lymphoid tissue-inducer cells (LTi), as well as other cells, specifically DCs, NKs, or CD8+ T cells, leading to the secretion of factors that are essential for high endothelial venule (HEV) formation (which mediate lymphocyte trafficking to lymph nodes), immune cell recruitment, and cell retention. Together, these aforementioned factors recruit and activate the LTi cells [31,32,33,34,35,36,37,38,39,40]. All these stages, from antigen absorption to cancer cell death, are considered as parts of the cancer immune cycle that will be discussed in detail in the following section.

**Figure 1:**
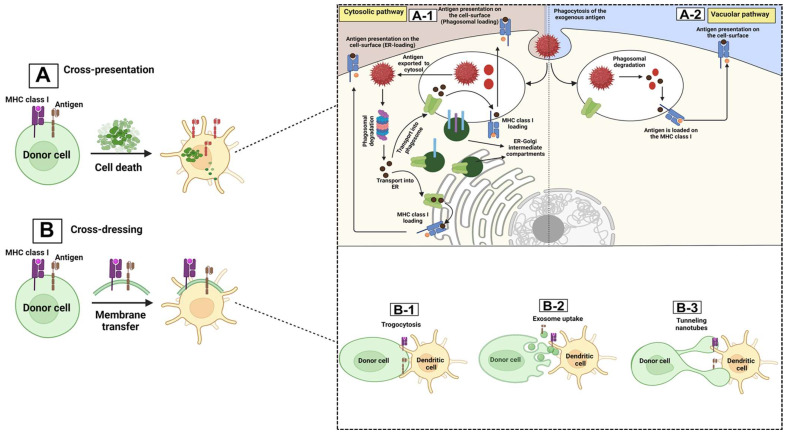
APC presentation mechanisms. tumor microenvironment and its components: TME contains a wide range of antigen cross-presentation processes that can be mediated by dendritic cells either through the canonical/cross-presentation pathways (**A**), or through the non-canonical/cross-dressing path (**B**). The cross-presentation mechanisms are mediated in two ways: cytosolic or proteosome degradation (**A-1**) and through the vacuolar pathway (**A-2**). In the cytosolic pathway, antigens that stem from either endosome or phagosome structures move toward the cytosol, forming acidic cytosolic proteosomes that cleave the antigens into shorter peptides. These peptides have two fates: (1) they are transported to the endoplasmic reticulum (ER) for further modifications. The modified antigenic peptides are then loaded on MHC class I molecules and move to the cell surface. (2) the cleaved peptides return to phagosomes/endosomes prior to loading on the MHC I and moving to the cell surface. In the vacuolar pathway, the aforementioned events related to antigen loading on MHC class I occur in the phagosomes or endosomes, which is followed by the moving of the antigen-loaded APCs (DCs) toward the secondary lymphoid organ (SLO) to activate T cells [13,41,49,50,51,52,53]. In the non-canonical/cross-dressing path, the antigen–MHC complex is formed on another cell and is then transformed to the APCs, such as DCs, and the DCs would finally be able to activate the related effector T cell via different pathways: trogocytosis (**B-1**), exosome uptake (**B-2**), and tunneling nanotubes (**B-3**) [59,60,64,65,66,67]. In Trogocytosis, the membrane patch, including the plasma membrane and cytosol from one cell (donor), is transformed to the other cell (trogocytic) [58,59]; exosome uptake by APCs depends on the ability of the exosomes (small membrane-based vesicles formed during the endocytosis process) to transfer particular materials (that can not only be further degraded and reprocessed by APCs for presentation on the MHC molecules, but can also be considered as functional MHC–peptide complexes [59,68]); the tunneling nanotubes are long protrusions derived from cell membranes that not only facilitate the exchange of cell surface molecules and cytoplasmic contents but also can mediate cross-dressing between remote DCs through the transferring of MHC molecules between distant cells [59,69].

Among the various APCs present in the TME, DCs are the most potent APC compared with B cells and macrophages. These cells mediate the antigen priming-related processes through two general mechanisms: canonical (cross-antigen presentation) and non-canonical (cross-antigen dressing) pathways [41,42]. The canonical pathways are more commonplace and are based on the type of the antigenic proteins (exogenous/endogenous). APCs (like DCs) can drive the canonical antigen presentation mechanism via two major pathways: (1) the cytosolic or proteasome degradation path (specified for endogenous protein presentation), during which the endogenous antigenic proteins, with either a proteosome or a phagosome origin, are cleaved by the cytosolic proteasomes of the DCs to generate peptide fragments, which then become presented by MHC-I molecules and activate the effector T cells against tumor cells, in particular antigen-specific CD8+ cytotoxic T lymphocytes (CTLs) [43,44,45,46], or (2) the vacuolar or endocytosis path (specified for exogenous antigenic protein presentation), during which DCs take up the exogenous antigenic proteins via the endocytosis process to form special vesicular structures called endosomes, which will then be fused with lysosomes, where the lysosomes’ low pH degrades these antigens to peptide fragments, which are then presented on MHC-II molecules, and activate the CD4+ T cells, leading to CTL activation, function, and survival [13,23,41,47,48,49,50,51,52,53,54,55]. Apart from the above-mentioned canonical pathways, several papers have demonstrated the presence of non-canonical/cross-dressing pathways through which the APCs such as DCs do not present the antigen themselves. Instead, the antigen–MHC complexes from the other adjacent DCs or tumor cells (donor cells) get transferred to the APCs, such as DCs (receiver cells), though various mechanisms, including trogocytosis, exosome uptake, and tunneling nanotubes [49,56,57,58,59], and activate the related effector T cells without further antigen processing stages [60,61,62,63,64,65,66]. These processes are shown in Figure 1.

## 3. Cancer Immune Cycle

**Figure 2:**
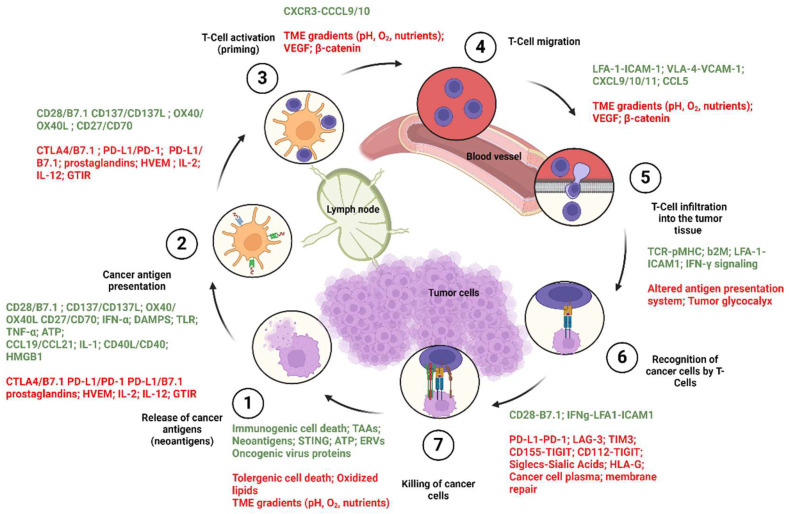
Caner immune cycle phases and regulation. Cancer immune cycle phases are regulated by a wide spectrum of cytokines and chemokines, some of which stimulate the cancer immune cycle to kill the cancer cells, whereas some cytokines act as inhibitors and down regulate the processes. The stimulatory cytokines work together to mediate the T cell activation. Activated T cells go the TME and induce tumor killing. Each phase is regulated by a wide range of cytokines and other molecular factors, as shown in green for inducers and red as inhibitors.

The cancer immune cycle includes a series of repeated and amplified phases, each of which are mediated by specific cytokines and chemokines which will lead to effective anti-cancer immune response and cancer cell death. Theses stages are as follows: (1) In the first step, the neoantigens that were generated during the tumor formation process are released from the dead tumor cells, which are then carried by the DCs to the adjacent draining lymph node (DLN). (2) The second stage starts with DCs presenting the acquired antigen to T cells through MHC-I and MHC-II molecules to form the MHC-I and MHC-II–antigen complexes, through the cross-presentation pathways discussed earlier. (3) Effector T cells can then recognize the antigen and become activated. (4) Antigen-recognizing tumor-specific T cells present in the DLN, express specific chemokine receptors as well as cell adhesion molecules required for the migration of T cells and their infiltration into the tumor tissue. By virtue of these expressed molecules, T cells leave the DLN and move toward the tumor tissue via the blood stream. (5) This is followed by T cell infiltration into the tumor tissue and (6) the recognition and binding of the MHC-I–antigen complex by virtue of the T cell receptor (TCR), which stimulates the secretion of various cytokines from the DCs that finally activate the T cells. (7) These processes work together to eventually kill the cancer cell [67,70] through various mechanisms, including direct tumor lysis and degranulation [71], antibody-dependent cellular cytotoxicity [72], and/or complement-dependent cytotoxicity [73,74,75]; however, a new pathway for the cancer-killing action of the T cells have been reported recently, which is independent of the antigen presentation by the MHC-I and its recognition by T cells [76]. Considering that additional neoantigens are released upon cancer cell death, causing the immune reaction and continuing the cycle again from the first phase where the neoantigens are upregulated by the cytokines, this mechanism is named cancer immune cycle, the steps of which are shown in Figure 2 in more detail. The cancer immune cycle becomes malfunctioned in cancer patients, as at least one of these steps is defective [67,77]. With this in mind, one of the effective ways to treat cancers would be to develop therapeutic vaccines that can target the cytokines in the cancer immune cycle (Figure 2).

## 4. Escaping from the Cancer Immune Cycle

The host immune system constitutes a surveillance part and a protective part; the immune surveillance system constantly inspects the body and boosts antitumor immune responses in order to identify and destroy any existent tumor cells and finally to prevent cancer progression [78,79,80,81]. In general, cancer cells undergo various genetic and epigenetic modifications, leading to the generation of specific antigens; these antigens then stimulate T cells to recognize and kill cancer cells; however, as tumor cells grow, they start to develop mechanisms known as “cancer immunoediting” to escape this host immune surveillance system; thus, the immune system is not able to eliminate these cancer cells [79,82,83]. On the other hand, looking at the protective part of a normal immune system, it consists of specific protein molecules called “immune checkpoints” that are present on the surface of immune cells (including T cells), and their corresponding ligand receptors, which are present on the cancer cells. These immune checkpoints induce inhibitory signals (using the mono-tyrosine-based signaling motifs, in particular, immunoreceptor tyrosine-based inhibitory and switch motifs) that prevent the generation of any strong immune response signals that destroy healthy cells in the body. In this way, immune checkpoints tend to protect normal cells [84,85,86]. PD-1, CTLA-4, LAG3, TIM3, BTLA, and TIGIT are some of the common immune checkpoints that mediate tumor cell recognition by T cells; when the immune checkpoint proteins present on T cells’ surfaces recognize and bind to the tumor cells’ receptors, they send an “off” signal to the T cells and hence prevent the eradication of cancer cells by the immune system. With this in mind, some cancer cells tend to facilitate cancer growth and metastasis by upregulating negative signals via cell surface immune checkpoint molecules and inhibiting T cell activations [87,88], while some other tumor cells may activate immunosuppressive leukocytes (such as eosinophils) to create a TME that is unable to respond to antitumor immune molecules well [89]. Moreover, some tumor-intrinsic genes, including YTHDF1, degrade the MHC-I complex molecules, resulting in immune evasion [90]. According to the mechanisms of action of the cancer vaccines as well as the cancer immunity cycle discussed earlier, some of the key factors for a successful cancer vaccine design are the selection of the appropriate tumor antigen to stimulate effective T cells, the achievement of a sufficient antigen concentration in APCs in such a way as to activate them, as well as the inducement of durable immunogenic responses by activating the effector T cells, i.e., CD4+ and CD8+ [23]. In this respect, the selection of the right antigen along with its delivery method would be of high importance, which will be discussed in detail in the following subsections.

## 5. Tumor Antigen Classifications

Tumor antigens are any antigenic substances generated in tumor cells that trigger an immunogenic response and serve as biomarkers for tumor recognition that can be used to develop novel therapeutic cancer vaccines. Tumor antigens can appear in phases pertinent to protein synthesis and degradation [82,91]. On the basis of the expression patterns of the HLAs, tumor antigens can be classified into two general groups: tumor-associated antigens (TAAs), in which the antigen is presented by the HLAs that are only expressed on tumor cells (mainly HLA class I), and tumor-specific antigens (TSAs, or neoantigens), in which the antigen is presented by the HLAs that are expressed not only on cancer cells, but also to normal cells [28,92].

**Figure 3:**
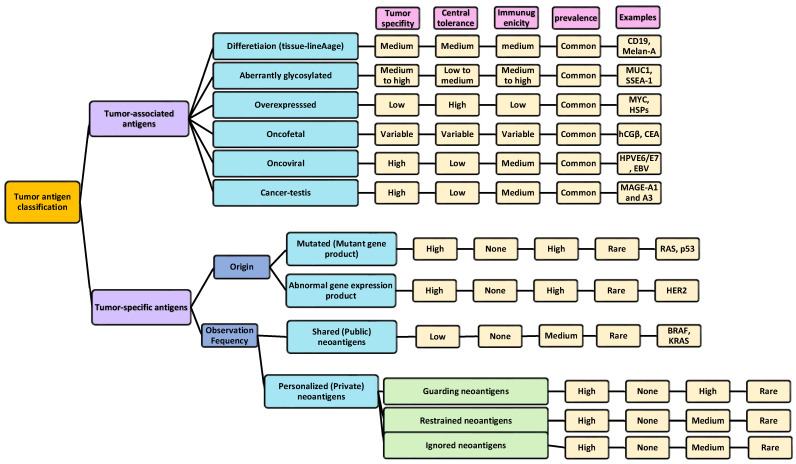
Tumor antigen classifications. In general, tumor antigens are classified as tumor-specific and tumor-associated antigens, each divided into several categories. Each category is compared in terms of (1) tumor specificity (refers to the degree to which a particular immune response targets and interacts specifically with tumor cells or its antigens while sparing normal cells; hence, a high tumor specificity is desired), (2) central tolerance (mechanisms by which the immune system recognizes the self-antigens from cancer antigens and eliminates cancer cells during their development within the body without mounting immune responses against the self-antigens. Thus, a high central tolerance is desired, as it means that the immune system exhibits a strong level of tolerance towards self-antigens, including those present on normal cells and tissues, and can recognize them better), (3) immunogenicity (indicates the ability of cancer cells to stimulate an immune response from the host immune system. This immune response can involve the activation of immune cells, and the production of antibodies against tumor-specific or tumor-associated antigens; so, high immunogenicity is a desired factor), and (4) prevalence (this shows how common or rare the occurrence of tumor antigens is in patients) [25,26,54,93,94,95,96].

Based on the molecular structure and sources of the antigens, TAAs can fall into one of these categories: differentiated (tissue-lineage), oncofetal, cancer–testis, aberrantly glycosylated and expressed, overexpressed, as well as oncoviral antigens. On the other hand, TSAs are classified, according to the frequency of observations, into shared (public) and personalized (private) neoantigens; shared (public) neoantigens arise from alterations that occur specifically in tumors which are observed across other patients/different malignancies, while their personalized (private) counterparts are those originating from tumor-specific alterations that are less likely to occur across other populations/malignancies; thus, personalized neoantigens are patient-specific. To date, a few numbers of public neoantigens have been recognized, whereas the private neoantigens usually originate from non-recurrent driver/passenger mutations and comprise most of the known neoantigens [25,26,54,93,94,95,96]. Furthermore, based on the source from which the antigen is derived, the antigen can be canonical (derived from the protein-coding genes), or non-canonical (derived from non-protein coding genes); in the canonical antigen, the antigen is expressed within the open reading frames (ORFs) of the protein-coding genes [93], such as the overexpression of numerous cancer-related genes [60,65,94], including p53 [97,98,99,100], cancer/testis antigens (CTAs) [100,101], and the human telomerase reverse transcriptase [102,103]. Non-canonical antigens are expressed outside of the ORFs [104], and can stem from alterations of the antigen at various levels, such as genomic, epigenomic, proteomic, transcriptomic, translational, and antigen-processing levels (intronic retention, alternative splicing, codon read-through, and noncanonical/non-AUG translation initiation levels) [93,100,105]. The tumor antigen classifications along with their properties are summarized in Figure 3.

Apart from the type of the antigen, determining an efficacious method for the delivery of the tumor antigen to the APCs would not only make antigen-mediated APC targeting more selective and induce T cell activations, but it would also decrease systemic toxicity. Since different types of antigens have various physical properties, inducing an optimum immune response would depend mostly on the selection of an appropriate delivery system [23]. Some of the major delivery methods are using cells, antigens, peptides, nucleic acids, and viral-based ones, each of which will be discussed in detail in the following sections.

## 6. Different Cancer Vaccine Platforms

### 6.1. Peptide-Based Vaccines

In peptide-based cancer vaccines, usually 20–30 amino acids are used to make a wide range of peptides for activating the immune system of patients, enabling them to recognize and kill the tumor cells by enhancing the T cell-mediated immune responses specific to a particular tumor, i.e., CD8+ and CD4+ T cells via the MHC class I and II molecules, respectively [106,107]. These peptides usually belong to one of the TAAs or TSAs (including cancer/testis antigens and neoantigens) that are used for designing personalized vaccines. Peptide-based cancer vaccines can not only activate both B cells and T cell-mediated immune responses, but also induce long-lasting tumor-killing effects [108]; however, to elicit an efficient antitumor T cell response, cancer vaccines usually deliver a mix of tumor antigen peptides including TAAs and TSAs. The identification and discovery of tumor antigen peptides have been discussed in other reviews [54,93,109]. Synthetic long peptides (SLPs) are stronger than short peptides in activating T cell responses because SLPs need to be processed by the APCs and can activate both cytotoxic CD8+ T cell and CD4+ T helper cell responses [110,111]. Due to low immunogenicity, peptide-based vaccines are usually formulated with immune adjuvants. Adjuvants have been licensed by the FDA and EMA for humans, include aluminum salts, MF59, adjuvant systems, and CpG 1018 [112]. Other adjuvants under investigation are polyinosinic-polycytidylic acid stabilized with polylysine and carboxymethylcellulose (poly-ICLC), glucopyranosyl lipid A, Imidazoquinolines, CpG oligodeoxynucleotides, cyclic dinucleotides, etc. [113]. To further increase the immunogenicity of peptide antigens, heteroclitic peptides, which are modified versions of peptides that have been altered (by replacing amino acid residues in the epitope sequence that have similar biochemical properties, overall structure, and function compared to the original amino acid sequences; this is known as conservative amino acid substitution) to enhance their binding affinity to the MHC molecules; in this way, they can induce an enhanced immune response against specific antigens, making heteroclitic peptides a potential tool in vaccine development and immunotherapy for diseases such as cancer [114,115,116,117]. Considering the short half-life and poor stability of the free peptides in the body, tumor antigen peptides are usually incorporated into other delivery systems. Poly lactic-co-glycolic acid (PLGA) nanoparticles and liposomes are two representative delivery systems of antigen peptides and adjuvants because of their proven safety [118,119]. A comparative study by Varypataki, Jiskoot et al. showed that SLP-loaded PLGA nanoparticles and cationic liposomes are more potent for stimulating the T cell responses in vivo than squalene or Montanide-based emulsions [119]. Both vehicles can protect the peptides from degradation and promote dendritic cell uptake and lymph node transport.

In the last decade, liposomal vaccines evaluated in clinical trials include Tecemotide, DepoVax, ISCOMATRIX, Lipo-MERIT, etc.; however, none of them improved the patients’ survival [120,121]. In addition to synthetic nanoparticles, dendritic cell-derived exosomes (DEXs) could play an important role in tumor immunology by transferring MHC/peptide complexes to other immune cells and stimulating T and NK cells directly or indirectly [122,123]. DEXs loaded with antigen peptides have been assessed as cancer vaccines in clinical trials, but failed to generate adequate adaptive immunity in patients with advanced cancer [123]. Nevertheless, DEXs still hold the promise of being a part of combination therapies.

### 6.2. Recombinant (Pathogen) Vaccines: Viral and Bacterial-Based Vaccines

There are three major classes of recombinant viral/bacterial vaccines: (1) inactivated vaccines (that use killed virus/bacteria that has been cultured in the lab) [124,125], (2) live attenuated vaccines (in which the virus/bacteria is being weakened but not completely killed) [126,127], and (3) subunit vaccines (in which a portion of the virus/bacteria-like protein is used) [126,128,129]. All recombinant vaccines are based on administrating the recombinant genes (such as genes encoding TAAs, cytokines, or costimulatory molecules that are inserted into the viral/bacterial genome) using recombination/selection methods into APCs to stimulate the appropriate antitumor immune responses and by engaging both innate and adaptive immune systems. Viral/bacterial-based vaccines can provide effective and long-lasting immune responses [130,131,132,133,134,135]. These vaccines target the APCs and initiate immune responses through two major mechanisms: (1) the indirect infection of the APCs, which works through cellular damage mediated by viral infection to send danger signals and as well as costimulatory molecules to activate the APCs of bone marrow [130,136,137], and (2) the direct infection of the APCs, which is based on the processing of the antigens in the MHC pathways. The latter mechanism facilitates recombinant viral vaccine modifications for enhancing the antigen presentation [130,138,139]. Some of these modifications are based on expressing the genes encoding the minimal level of MHC class I-restricted peptides [140], inserting endosomal/lysosomal sorting signals into the gene encoding antigen [12,141], as well as using poxviruses to activate T cells [142,143,144], or to be used as a vector to carry specific costimulatory molecules or cytokines [145]. One of the recent effective bacterial-based cancer vaccines was developed by Wu et al.; these researchers used attenuated flagellated bacteria (strain of *Salmonella typhimurium*) coated with positively charged dendrimer nanoparticles with the ability to bind to negatively charged antigens, and the bacterial had become less immunogenic via gene mutations [146].

### 6.3. Cell-Based Vaccines: Dendritic Cells (DCs), Stem Cells, and Chimeric Antigen Receptor (CAR) T Cell Therapy

Therapeutic cell-based vaccines are based on the in vitro activation of the APCs (like NK cells or DCs) by the viral peptides, genes, or by using genetically modified tumor cells (killed tumor cells). In this regard, the cell-based vaccines can be classified as tumor cell vaccines and immune cell vaccines [147]. In the tumor cell vaccines, the whole tumor cell is used as the source of the vaccine, which contains whole TAAs, including the CD4+ and Cd8+ T cells’ epitopes. Whole-cell cancer vaccines are currently in clinical trials. Using whole tumor cells as a vaccine that has all the possible antigens in it rather than protein/peptide tumor antigens not only eliminates the need to identify the ideal target antigen; in addition, several tumor antigens can be targeted at once, which would then induce further immune responses to more tumor cells [148,149]. However, there is still a need for a stimulus/stimulatory factor(s) to provoke the antigen absorption process by the APCs to recruit cells from innate and adaptive immune systems. With this in mind, cell-based vaccines have been modified either genetically or via irradiation in such a way as to be able to secrete cytokines without further proliferation in the host [147,150,151]. Most of the recent developed cancer vaccines are based on using whole cells like DCs, which affect the function of the cells in the immune system. The importance of DCs in antigen uptake and presentation processes, as well as T cell activations, which are mediated by a wide spectrum of receptors present on DCs’ surfaces, including those for antigen uptake, antigen presentation, costimulatory molecules, cytokines receptors, receptors for environmental sensors, cytokine production-related receptors, as well as chemokine receptors, have turned DCs into the most commonplace immune cells used in developing immune cell-based cancer vaccines [12,22,39,49,147,152,153,154]. To improve the efficacy of DCs in antigen absorption and T cell activation, researchers have started to use stem cells to develop better cell-based cancer vaccines. The application of stem cells in the realm of cancer vaccines started with embryonic stem cells (ESCs) [155]; considering that ESCs are usually obtained from an unrelated donor, they express a mismatched MHC and minor histocompatibility (miH) antigens (which are peptides derived from normal self-proteins that, in humans, are presented by HLA), and if transplanted in the host, they will cause alloimmune responses [156,157,158]. Although ESCs express a low amount of HLA-I [159,160,161,162,163] and almost no HLA-II [162,163,164,165] and costimulatory molecules [162,164,165], this amount is sufficient to stimulate the cytotoxic T cell-mediated xenorejection of human ESCs [158,166,167]. Following the characterization of human ESC lines, and considering the ability of whole-cell vaccines to deliver multiple oncofetal antigens at once, along with their universal application to all patients regardless of their HLA type [156,168,169], researchers have started to apply these ESCs to whole-cell cancer vaccines to make ECS-based cancer vaccines. Using xenogeneic human ESCs as the plausible cancer vaccine to be tested on mouse and rat models, studies have found that human ESCs resulted in a moderate tumor killing effect, whereas in the case of using allogeneic or autologous ESCs, they observed more potent tumor suppressive effects [169,170]. However, considering that human ESCs were injected into mice, there was the possibility that the aforementioned immune responses were due to the incompatibility of the MHC antigens between the human ESCs and mouse cells rather than the ESC lines [169]; moreover, the tumorigenicity induced by the ESCs hampered their usage as effective cancer vaccines for clinical applications [171,172,173]. These problems led researchers to shift their focus toward using induced pluripotent stem cells (iPSCs), as they share very common features with ESCs in terms of gene expression and epigenetic profiles [174,175,176,177,178,179]. However, iPSCs also have some level of tumorigenicity. Various methods have been reported to overcome their tumorgenicity when developing stem cell-based vaccines: the terminal differentiation or complete elimination of residual iPSCs from culture; interfering with tumor-progression genes to prevent tumor formation from the residual cells; and tumor detection and elimination after its initial formation in the patient’s body [173]. In light of this, most of the recent work with iPSCs have used irradiation to remove the residual iPSCs in the culture and strongly prevent teratoma formation and further iPSC-mediated tumorigenicity [180,181,182,183]. Early studies working on iPSCs transfected to mouse colon cancer demonstrated that although the iPSCs were able to induce cytokines in response to the cancer cells, no tumor rejection was observed, indicating that iPSCs need modifications to be able to induce a strong immune response against tumor cells; for instance, considering that autologous iPSCs have more accurate tumor antigens compared with their xenogeneic counterparts, they can be a better option for developing anti-cancer vaccines than the xenogeneic ones, as they can minimize the alloimmunity; further, to enhance their immune responses against cancers, immunostimulatory adjuvants can be used with them (such as TLR9) [169]. Kooreman et al. used the same strategy to generate an iPSC vaccine against pancreatic ductal adenocarcinoma, in which the autologous iPSCs were irritated, followed by the addition of CPG (a type of TLR 9 adjuvant) to improve the immune response [169]. Another study developed autologous iPSCs from patients with T cell acute lymphoblastic leukemia and were loaded in DCs; this showed efficacy in suppressing acute lymphoblastic leukemia cancer [184]. In a recent study, iPSC-derived exosomes were incubated with DCs (dendritic cells) and their antitumor effects were explored in murine melanoma models; according to their results, the DC+ exosome vaccination significantly inhibited lung metastasis in in vivo models, induced long-term T cell responses, and did not alter the viability of normal cells and mouse viscera [185]. In the same way, another group prepared a nanostructure by combining the iPSCs and DC exosomes that contained the anticancer drug doxorubicin; this improved the in vivo efficacy of chemotherapy drugs as well as the antitumor immunity [186]. Apart from iPSCs, researchers have used inactivated cancer stem cells (CSCs) to develop cancer vaccines [187]. Chimeric antigen receptors (CARs) are recombinant protein receptors that have been engineered in such way as to enable T cells to target a specific antigen in order to generate an antitumor immune response and kill specific tumor cells. The general structure of these receptors are made up of three major domains: (1) an extracellular domain specified for selective binding to a particular tumor antigen, (2) a transmembrane domain, and (3) a intracellular domain; together, these domains facilitate T cell-mediated tumor death by providing T cell signals that are necessary for their activations and for attacking the tumor cells [188,189,190,191]. One of the examples of CAR-T cell therapy is based on using genetically modified autologous T cells expressing CD-19. The therapy reprograms the patient’s own T cells via a transgene that encodes the CAR and is able to recognize and destroy any cells (normal and malignant) that express the CD-19, in a way that, after binding to CD19-expressing cells, the CAR sends a signal that enhances the T cell expansion, activation, and target cell elimination, along with the persistence of the drug. The aforementioned mechanism can be seen in two of the current FDA-approved drugs based on CAR-T cell therapy, i.e., Tisagenlecleucel (used to treat acute lymphoblastic leukemia) and Axicabtagene ciloleucel (used for treating large B cell lymphoma) [192,193]. However, there are some limitations to overcome: there is the possibility of antigen loss, so that patients treated with CAR-T cells may partially express the antigen or may not express it at all [194,195,196]; another problem is the possibility of the expression of the tumor antigen by normal cells [197]. Although the combination of checkpoint inhibitors and CAR-T cell therapy is a new treatment option, this treatment may still be unable to induce efficient T cell infiltration and may lead to cytokine-mediated toxicities that have been reported in several CAR-T cell therapies [198,199,200,201]. This requires looking for a novel method to optimize the CAR-T cell therapy-based cancer vaccines.

### 6.4. DC Subsets and Their Roles in Priming and Activating T Cells

**Figure 4:**
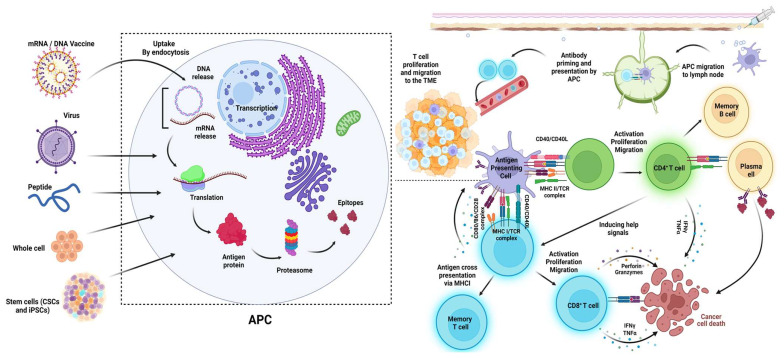
Cancer vaccine platforms and their mechanisms of action. All of the discussed vaccine platforms tend to be up taken by the antigen-presenting cells to finally induce and enhance the T cell-mediated pathways of killing cancer cells [404].

DCs originate from macrophage–DC progenitors (MDP) in bone marrow and generate common DC progenitors (CDP) that then differentiate into the DCs [154,202], which comprise various types of immune cells that, based on their phenotypes, ontogenetic features, distribution in tissues, as well as transcriptional-related characteristics, are divided into three major groups: classical/conventional DCs (cDCs) (that include cDC1s and cDC2s), plasmacytoid DCs (pDCs), as well as monocyte-derived DCs (moDCs) [203,204]. Each of these DC groups secrete specific types of cytokines that are specialized for priming and activating various classes of effector T cells and regulating particular stages of the cancer immune cycle; thus, in this way, they can affect the result of an immune response in different ways [49,202,203,205]; for example, cDC1s are specialized for antigen priming as well as their cross-presentations to the CD8+ T cells, followed by their recognition via MHC I signaling [202,206,207,208,209,210,211,212]. On the other hand, cDC2s are mostly involved in the cross-presentation of the antigens to CD4+ T cells and their recognition through the MHCII path, promoting Th1, Th2, and Th17 polarization [202,211,212,213,214,215,216]. According to single-cell analysis, a further level of complexity in DCs has been reported via the identification of various types of cDC2 subsets [203,217,218]. pDCs produce type I interferons (IFNs) that are engaged in antiviral and antitumor immune responses [202,212,216,219,220]. Finally, moDCs, which are stimulated by inflammation, become differentiated and recruited to inflammatory parts of the body, such as the TME [216,221,222,223,224]. Prior to encounters with the antigen, DCs are immature and characterized by a high expression of MHC-II inside the cell, low expressions of co-stimulatory molecules and chemokine, and cytokine receptors [225,226,227]. However, these immature DCs uptake the antigen via the cross-presentation process or cross-dressing, and become mature DCs through various pathways, in particular receptor-mediated endocytosis [228,229,230,231]. Due to the presence of various types of receptors on the their surfaces (Figure 4), DC maturations can be stimulated by different factors, ranging from monoclonal antibodies (mAb) to DCs modifications, and the physiological alterations in DCs occur during their maturations, enabling DCs to secrete a wide range of stimulatory cytokines and other chemical molecules to block the inhibitory signals and increase co-stimulatory molecules, cytokine production, and antigen presentation [232,233,234,235,236]. DCs then process and present tumor antigens derived from the vaccinating cells to the effector T cells (CD4 and CD8) via the formation of antigen–MHC complexes on the DCs, and T cells bind to this complex with their T cell receptors (TCRs) [17,234,235,237]. During maturation, DCs undergo physiological alterations, leading to the incremental expression of surface MHC I and MHC II molecules [238,239], co-stimulatory molecules (such as B7-1/CD80, ICAM-1/CD54, LFA-3/CD58, and Tropomodulin1) [240,241,242,243], chemokine receptors [244,245], and cytokine secretions [246,247,248,249], that together regulate the T cell response. Furthermore, DC maturation results in a reduction in the pH of the endocytic vacuoles, leading to proteolysis, the transport of peptide–MHC molecules to the cell surface, and a reduction in the capacity for antigen capture [250,251,252,253,254,255]. Following the upregulated expressions of various stimulatory molecules/receptors, the DCs migrate toward the draining lymph nodes to interact with the T cell and induce the immune response to finally present the tumor antigen derived from the vaccinated cells to the effector T cells (CD4 and CD8) via the formation of antigen–MHC complexes so that T cells can bind to these complexes present on the surface of the DCs with their receptors (TCRs) and become activated; this process leads to tumor killing [17]. If the CD8 T cells are activated efficiently, with addition of other traditional cancer therapy methods, such as monoclonal antibodies, chemotherapy, and radiation therapy, they all can work together synergistically to improve the efficiency of T cell-mediated tumor-killing effects [256,257,258,259,260]. With this perspective, T cell activation regulation is one of the key factors to be considered when developing cancer vaccines. T cell activation is modulated by a wide range of other factors and signals produced by the activated DCs [261], agonist antibodies [262,263], co-stimulatory molecule receptors [264,265], and co-inhibitors (immune checkpoint inhibitors) [84,266]. However, in order to maintain immune homeostasis and self-tolerance, as well as to reduce/prevent inflammation and autoimmunity diseases, it would be necessary to inhibit the effects of stimulatory signals when needed. In light of this, specific molecules, including (1) a heterogeneous Foxp3 expressing a subset of CD4+ T cells known as regulatory T cells (Tregs) that have immunosuppressive properties [267,268,269,270], and (2) other suppressive immune cells, such as myeloid-derived suppressor cells (MDSC) [271,272,273], act and suppress by secreting various inhibitory cytokines and molecules (such as TGF-β, IL-10, and IL-35) [257,274,275,276,277]. Chemotherapy and radiation, if used at immunomodulatory doses, could inhibit the T cell activation [278,279,280,281,282].

### 6.5. Nucleic Acid-Based Vaccines: DNA and mRNA Vaccines

Nucleic acid vaccines are based on using either DNA or mRNA to deliver genes to the host APCs to encode the tumor antigens and produce antigen proteins so that the expressed tumor antigens induce appropriate immune response to kill/inhibit cancer cells [283].

#### 6.5.1. DNA-Based Cancer Vaccines

The history of using DNA cancer vaccines goes back to 1990 when Wolff et al. studied the effects of the direct injection of naked DNA to murine muscles, which resulted in the expression of their corresponding proteins [284]. And in 1998, the first human trials of a DNA vaccine were reported, which demonstrated the efficiency of DNA vaccines in treating immunodeficiency virus type 1 (HIV) [285]. Cancer DNA vaccines are based on using bacterial plasmids that encode the tumor antigens to activate both innate and adaptive immune responses. In order for the DNA vaccines to be functional, they need to enter to the cell nucleus to be transcribed into mRNA; then, they are transported to the cytoplasm to be translated to the encoded antigens, followed by antigen processing and presentation to CD8+ T (via MHC I) and CD4+ T (via MHC II) cells to activate particular immune responses [286,287,288,289]. The mode of action of DNA vaccines is the activation of adaptive and innate immune systems [290]. Regarding the adaptive immunity activation-based mechanisms, there are three major pathways: (1) the direct insertion of DNA into a somatic cell, such as a muscle cell, followed by translating to antigens, and their direct presentation to the cytotoxic CD8+T cells via the MHC-1 molecules [291]; (2) the releasing of the DNA-encoded antigen in somatic cells through secretion or via apoptotic bodies, followed by phagocytosis and the processing of the released peptides by APCs and their cross-presentations to the CD4+ T cells by the MHC II molecules [291]; and (3) the direct transfection of DNA into the APCs to generate antigens (which would be endogenous antigens). These endogenous antigens are then processed and presented to CD8+ T and CD4+ T cells via MHC I and MHC II molecules, respectively, to induce adaptive cellular immunity (via activation of CD8+ T cells followed by their differentiation to CTLs) as well as humoral immunity (by activating the CD4+ T cells); this direct transfection of DNA into APCs, which mainly takes the form of intradermal delivery, is a momentous pathway for DNA-based cancer vaccines [292]. Turning to the innate immunity activation pathways mediated by DNA vaccines, there are a wide range of factors that regulate the aforementioned pathway, such as CpG (cytosine phosphate guanosine) dinucleotides, which are immunostimulatory motifs within bacterially produced plasmid DNA [293]. These are involved in stimulating innate immunity activation by interacting with one of the key innate immunity stimulators, i.e., Toll-like receptor 9 (TLR9). This is followed by thTLR9 recognizing unmethylated CpG motifs in bacterial DNA, resulting in the triggering of the TLR-mediated signaling pathway of macrophages, dendritic cells, and B cells, which involves activations of the NF-κB, IRAK, and MyD88 signaling pathways to produce proinflammatory cytokines, chemokines, and immunoglobulins [113,294]. Furthermore, DNA itself activates the STING signaling pathway, which is the major pathway controlling the DNA signaling cascades, which occur in cytoplasm independent of TLR. In vivo studies have confirmed that DNA vaccines cannot induce a robust adaptive immune response in the absence of the STING path [113,295]. DNA vaccines offer various advantages, including being highly specific and safe, encoding a wide range of antigens, having low production costs, as well as easy transport and storage; moreover, DNA vaccine have a lower risk of insertional mutation and DNA rarely binds to host chromosomes [296,297,298]. Furthermore optimized DNA vaccines have been efficient in preclinical studies [299,300,301,302]. However, because of their poor immunogenicity, DNA vaccines have gained little progression in clinical trials [303,304]. There are several optimization strategies to tackle the poor immunogenicity problem, including the optimization of plasmid elements (such as the Kozak sequence, intron, and species-specific codons) [305,306], a powerful promoter sequence for an efficient transcription (such as modified viral cytomegalovirus promoters) [306,307,308], using specific adjuvants (such as cytosine–guanine dinucleotide (CPG) motifs, polymers, nanoparticles, liposomes, and small molecule agonists) [305,306,309], and finally, modifying the design of tumor antigens [305,306].

#### 6.5.2. mRNA-Based Vaccines

In vitro transcribed mRNA vaccines are the very early versions of mRNA vaccines developed in 1984 using an in vitro transcribed system containing a plasmid DNA template, RNA polymerases, along with other main components [310]. Although at first mRNA vaccines were not developed for therapeutic purposes, early research, including the first in vitro (using DCs that were pulsed with RNA) and in vivo (in mice) studies pertinent to the mRNA-based cancer vaccine back in the 1990s [311], paved the way for using mRNA vaccines for treating diseases, including cancers. Subsequent research that focused on delivering mRNA into the cells using liposomes further confirmed the therapeutic efficiency of mRNA-based vaccines [312], as mRNA vaccines bring a wide spectrum of benefits, such as tolerability (side effects are controllable and temporary) and the lack of a need for genome integration (because unlike DNA, there is no need for the mRNA to enter the cell nucleus); thus, the risk of insertional mutagenesis is eliminated. There is no need for the usage of any pathogenic/viral agents for developing mRNA-based vaccines; therefore, it is non-infectious. Furthermore, mRNA vaccines are degraded easily (which reduces risk of toxicity), providing humoral and cellular immunity, which are essential for antitumor responses. Additionally, mRNA vaccine production is fast and inexpensive [313,314,315,316]. There are four major types of transcribed mRNA: conventional mRNA, self-amplifying mRNA (samRNA), trans-amplifying RNA (tamRNA), and circular mRNA (circmRNA) [317,318,319,320]. The general structure of these in vitro transcribed conventional mRNAs are similar to natural mRNAs in eukaryotic cells, i.e., they are made up of a 5′ cap, 5′ and 3′ untranslated regions (UTRs), an open reading frame (ORF), and a poly(A) tail [320,321,322,323]. In spite of the advantages of such mRNA vaccines, some drawbacks, including the inherent instability of the mRNA, lack of good manufacturing practices, low protein expression efficacy, high immunogenicity, along with the difficulties related to the in vivo delivery of mRNA into cells, and off-target side effects as a result of repeating the injection dosage to maintain protein expression, have hampered its advancement as an effective therapeutic vaccine [314,324]. With this in mind, in recent decades, considerable efforts have been made to optimize mRNA-based vaccines by improving mRNA stability, reducing its in vitro and in vivo immunogenicity through chemical modifications, product purification, and sequence optimization, such as the 5′ end (autologous) or the 5′ cap (analogues) modifications, ORF modification by codon optimization, guanine plus cytosine (GC) content enrichment, maintaining the length of the 3′ poly(A) tail within 120–150 nucleotides, and adding chemically modified adenosines [325,326,327,328,329,330]. Another way to improve the stability and the protein yield was to develop other types of vaccines which are based on RNA rather than mRNA. For example, self-amplifying RNA (saRNA), trans-amplifying RNA (taRNA), and circular RNA (circRNA) have brought therapeutic benefits in the realm of cancer vaccines [320,331,332,333,334,335,336,337,338,339,340].

However, in order for mRNA vaccines to be used in clinical applications, they should be protected from enzymatic degradation, successfully delivered to the target cells, followed by endocytosis, and escape from endosomes to prevent premature degradation. The physicochemical properties of mRNA complexes should be taken into consideration, as they affect the mRNA uptake mechanisms by the targeted cells [315,341,342]. With this perspective, the efficient delivery of mRNA to the targeted cell/tissue is necessary. To reach this goal, two major approaches have been developed: (1) ex vivo DC transfection via electroporation followed by re-infusion of the transfected cells [343,344,345], and (2) the direct injection of mRNA, with or without a carrier [322,345,346]. In the first approach, mRNA is loaded into the DCs through electroporation (to achieve optimized ex vivo transfection without using any carriers). Upon generating transfection DCs, they would be re-infused to the patient to act as carriers as part of an autologous vaccine and induce immune responses. With the ability to initiate adaptive immune responses as well as anti-body responses (by presenting the intact antigen to B cells), DCs have gained considerable attention to be used as ex vivo and in vivo carriers in the realm of mRNA vaccine delivery [347,348,349,350,351,352].

In the same vein, intradermal and intranodal injections were efficient in providing in vivo immunizations [353,354]. On the other hand, physical methods (using electroporation [355] or a gene gun [356,357,358]) increase the mRNA uptake efficiency by the DCs, but are faced with major limitations that hamper their further development, such as increasing cell death, and confining accessibility to target cells [359]; furthermore, using a gene gun, for instance, demonstrated efficiency only in mouse models but not in human models or larger study scales; electroporation increased the immunogenicity (only in the case of a self-amplifying RNA vaccine) [315,358,360]. Using viral carriers for mRNA delivery has several drawbacks, making them inappropriate carriers; some of these drawbacks include poor in vivo efficacy, the possibility of stimulation of immune responses mediated by the vectors, already-existing immunity against viral vectors, and biosafety issues [133,361,362,363]. These drawbacks led scientists to look for other mRNA carriers, which resulted in taking advantage of nanoparticles, in particular the lipid and polymeric-based nanoparticles, to develop versatile, effective, and safe carriers for mRNA delivery [364,365,366,367,368]. Some of these lipid/polymeric-based methods are based on using protamine (cationic peptide) [369,370,371], cationic lipids [372,373], and polymers, including dendrimers and chitosan [374,375,376], as well as lipid nanoparticles [352,365,377], which are conjugated with other polymers like polyethylene glycol (PEG) to increase the stability. In the case of lipid carriers, cholesterol and other natural lipids present in the membrane have been applied to enhance the efficacy. The lipid-based delivery vector not only improves the efficiency of mRNA delivery and facilitates the selective targeting of organs and/or cells (as it would be possible by adjusting the ratio of various elements in the lipid nanoparticle) [352,364], but also, such lipid-based carriers induce an adjuvant effect, as reported in some of the recent studies showing that lipid nanoparticles induce strong in vivo immune responses, with stronger adjuvant efficacy than AddaVax (a commonplace vaccine adjuvant) [322,378,379,380]; furthermore, lipid nanoparticles can enhance the antitumor efficacy of mRNA cancer vaccines through activating the Toll-like receptor 4 (TLR4) signaling pathway [381,382]. mRNA vaccines can be administered through several routes, such as subcutaneous, intradermal, intranasal, intramuscular, intranodal, intratumorally, and intravenous delivery routes [383]. The ex vivo engineering of autologous DCs with mRNA has been considered as the preferred method for tumor antigen delivery, but most approaches used for developing mRNA vaccines have a tendency to use direct mRNA administration with lipid nanoparticle carriers [343,384]. mRNA-based vaccination is developed to either induce or enhance an effective antitumor immune response. Following the administration and cellular uptake by APCs, mRNA goes to the cytoplasm and undergoes antigen priming and MHC-antigen presentation cascades, leading to APC-mediated antigen presentation via MHC-I and MHC-II and CD8+ and CD4+ T cell activation. Apart from that, CD4+ T cells themselves can induce a humoral immune response through coactivating antigen-specific B cells, and these B cells can serve as APCs to conversely activate CD4+ T cells upon the presentation of antigens to the B cells via MHC class II [385,386,387,388].

### 6.6. Personalized Cancer Vaccines

Personalized cancer vaccines are another type of immunotherapy designed to target a patient’s specific cancer cells based on their unique genetic profiles to stimulate the patient’s immune system to recognize and attack cancer cells more selectively [389]. Based on the different cancer vaccine platforms discussed earlier, several types of personalized cancer vaccines have been developed and used in preclinical and clinical studies, such as personalized cancer vaccines based on peptides [106,390], whole cells [390,391], nucleic acids (DNA and mRNA) [390,392], and neoantigens [390,393]. There are several steps for developing personalized cancer vaccines: (1) a genomic analysis of the patient’s tumor (to identify tumor-specific characteristics, such as mutations, neoantigens, and other related characteristics that can be targeted by the immune system); (2) antigen selection (based on the genomic analysis, tumor-specific antigens are selected for inclusion in the vaccine); and (3) vaccine formulation (vaccines are formulated using different approaches, such as with peptides derived from tumor antigens, DCs loaded with tumor antigens, DNA or RNA encoding tumor antigens, neoantigens [394], or whole tumor cell lysates) [395]. Upon administration of a personalized cancer vaccine, antigens in the vaccine are presented to immune cells by the APCs and stimulate an immune response against the cancer cells bearing the targeted antigens. Cancer vaccines, both conventional and the personalized type, contribute to the growing field of cancer immunotherapy, with personalized vaccines showing promise in improving treatment outcomes for patients with specific tumor profiles, as this type of vaccine offers a highly targeted and individualized approach to cancer immunotherapy, compared to its conventional counterpart that may target common antigens or cancer-associated antigens across broader patient populations. Personalized vaccines are designed to target the patient’s specific cancer cells based on the tumor’s genetic and antigen profile and stimulate the immune system to recognize and attack those specific cancer cells, while conventional cancer vaccines may target common antigens shared by several cancer patients or antigens associated with certain types of cancer but are not personalized to an individual’s tumor. Additionally, the antigens for personalized cancer vaccines are selected based on a genomic analysis of the patient’s tumor and neoantigens, whereas the targeted antigens in the conventional cancer vaccines are more broadly expressed across cancer cells of a particular type or could be associated with cancer but not specific to an individual’s tumor. Considering the production process, the personalized cancer vaccines have a customized production process, (sequencing the patient’s tumor DNA/RNA, identifying specific mutations, synthesizing, or selecting peptides or antigens based on these mutations, and formulating the vaccine); however, the conventional types are produced using standardized antigens or antigen sources that are not personalized to a patient’s tumor). Personalized cancer vaccines activate a targeted immune response against the patient’s specific cancer cells bearing the selected antigens; however, the conventional vaccines induce a generalized immune response against common cancer antigens or antigens associated with specific types of cancer. Personalized cancer vaccines are often used in precision medicine, where treatments are tailored to individual patients based on their unique characteristics. While personalized cancer vaccines offer promising potential in cancer therapy, there are also some challenges that need to be addressed; for instance, they require complex processes (such as genomic sequencing of the tumor, identification of specific antigens), and custom manufacturing of the vaccine for each patient. Considering that these processes are time-consuming, resource-intensive, and costly, these vaccines have limited accessibility among patient populations; this time-consuming problem may lead to other issues and these vaccines are not suitable for patients who require immediate treatment (those with rapidly progressing cancers), as the time required for genomic analysis, antigen selection, and vaccine production may delay treatment initiation [396]. Tumor heterogeneity is another challenge; considering the genetically heterogeneous nature of the tumors, they contain a mix of different cell populations with varying genetic mutations and antigen profiles, and thus, personalized vaccines may not target all relevant antigens present in the tumor, leading to potential escape mechanisms by cancer cells that are not targeted by the vaccine [397]. Furthermore, personalized cancer vaccines may face challenges in overcoming the immune evasion mechanism of the cancer cells, leading to limited efficacy in some cases; other barriers can be attributed to the limited clinical evidence along with other logistical challenges (including storage, transportation, and administration of the personalized cancer vaccines) [393,398,399,400]. Despite these challenges, ongoing research and advancements in cancer immunotherapy continue to improve the development and utilization of personalized cancer vaccines, with the aim of addressing these limitations and enhancing their effectiveness in treating cancer. The most effective approach has been the development of neoantigen-based personal cancer vaccines [394,400,401,402,403,404]. In general, all of the current cancer vaccine platforms have advantages, such as inducing both humoral and adaptive immune systems, long-term stability, flexibility, high immunogenicity, clinical safety, and etc. [106,187,324,364,405,406,407,408,409,410,411,412,413,414,415,416,417,418,419,420,421,422,423,424] along with disadvantages, such as antigen loss, low MHC expression, in appropriate APC uptake and antigen presentation, and etc. [106,201,287,363,383,411,412,416,418,419,422,423] that are summarized in details in Table 1. 

## 7. Combining Artificial Intelligence and Cold Plasma Technology as Novel Modality Tools to Develop Cancer Vaccines

Several major companies are currently the leaders of producing therapeutic cancer vaccines, including the following: Immatics, BioNTech, AstraZeneca, Memorial Sloan Kettering Cancer Center, Merck, Massachusetts General Hospital, F. Hoffmann-La Roche, Bristol-Myers Squibb, Regeneron Pharmaceuticals, and Novartis. In spite of numerous patents and the development of various cancer vaccines by the above-mentioned companies, there are several major challenges for the development of efficient and universal cancer vaccines, such as tumor variability in different people, the similarity of the tumor antigens to the body’s own antigens, as well as the possibility of cancer recurrence (caused by an immunosuppressive TME, or tumor heterogenicity) [390,425]. With this in mind, optimizing current cancer vaccines that can cover a wide range of tumor antigens, distinguish tumor antigens from the body’s counterparts, and prevent cancer recurrence is indispensable. As can be seen in Table 1, all of the vaccine platforms have their own particular pros and cons, and this has led scientists to optimize cancer vaccines by combining these different platforms with the aim of reinforcing the advantages and reducing the disadvantages, and to come up with better therapeutic cancer vaccines. Examples of such combination therapies include using lipid-based deliveries (liposomes, lipid nanoparticles, catanionic lipids), various polymers, in particular, PEG, to enhance mRNA stability, infusing polymers with the target cells, tumor specificity, amplifying the tumor antigen response, and reducing possible toxicities [426,427,428,429,430,431,432,433,434,435]. Furthermore, in order to overcome the immunosuppressive TME (which can prevent function and activation of immune cells necessary for destroying cancer cells [436,437]), researchers have combined the usage of mRNA vaccines with immune checkpoint inhibitors [328,438,439]. Tumor heterogenicity refers to the presence of genetically diverse subpopulations with different phenotypic profiles and leads to a diversity of genetic mutations. Tumor homogeneity can be seen between tumors or within the same tumor; tumor homogeneity makes it difficult to detect mutations that occur in subpopulations and has hampered the design of appropriate treatment strategies [440,441]. In terms of tumor heterogenicity, there are spatial (dynamic genome evolution through tumor progression) and temporal (tumor is made up of subclones with different genetic profiles which makes people with the same cancer and tumor subtypes respond differently toward treatments) heterogenicities and both should be overcome [324]. Some of the strategies were based on designing personalized mRNA cancer vaccines based on the variations seen in tumor regions via tissue multipoint sampling; these strategies are able target multiple antigens expressed across various tumor regions at the same time (to target the spatial heterogenicity) and monitor the progression of the disease, which can be followed by modulating treatment plans based on the results of monitoring (to tackle the temporal heterogenicity). However, these strategies and combination therapies make the vaccine design and administration routes more complex, and increase the cost and duration of the treatment time for patients [324]. With the extension of artificial intelligence (AI) applications in various sectors, including medicine, scientists have taken advantage of AI algorithms (such as MHC-binding prediction tools, quantification of mutated transcript expression, and clonality of the mutation, identifying tumor-specific T cell epitopes) to predict the tumor antigens and their properties based on tumor genomic data. Based on the likelihood of eliciting a T cell response, scientists can select some of the specific mutations as vaccine candidates. AI tools may enhance the accuracy of vaccine designs and overcome the challenges associated with the heterogeneity of tumors [442,443,444,445,446].

Using AI algorithms seems to be a promising tool that could help in addressing the major challenges of cancer vaccine development. Another possible technology that could be used along with the AI-related tools is cold plasma-related systems. Cold atmospheric plasma (CAP), also known as nonthermal or cold physical plasma, is a medium consisting of partially ionized gas(es) that provokes the generation of various reactive oxygen and nitrogen species. CAP has been applied in a wide range of industries, including medicine and in particular, in cancer therapy; CAP has shown promising results in destroying cancer cells as well as solid tumors by affecting various related mechanisms at the same time, such as inducing apoptosis, specifically in tumor cells but not in their normal counterparts, reducing cell migration, arresting the cell cycle at the S-phase, damaging the DNA, along with increasing the intracellular concentrations of ROS in the TME, reducing tumor immunosuppression, and improving antigenicity [447,448,449,450,451,452,453]. CAP has gained FDA approval to be used in cancer therapy [454,455,456]. However, this technology has not been used in cancer vaccine development yet. Based on the way that CAP has been applied in cancer therapy, we propose that CAP technology could be applied in cancer vaccine development directly or indirectly. The direct methods can include the direct exposure of cancer cells along with vaccine administrations (for example, simultaneous administration of lipid nanoparticle mRNA cancer vaccines and cold plasma exposure of the cancer cells), and the indirect method can include exposing either the cells in the culture medium (such as patient T cells in case of CAR-T cells, or iPSCs) or the culture medium alone to the CAP first and then growing the cells in the medium. This might help to reduce tumor heterogenicity by preventing genetic mutations with tumor progression, improving the selectivity of the therapy in killing cancer cells without affecting normal cells, and preventing tumor antigen expression by normal cells. Other methods may include trying to develop other new CAR-T cells, rather than CD19 and BCMA, which are able to recognize different tumor antigens, and applying cold plasma. Considering that CAP can enhance the tumor antigenicity (the degree of difference between cancer and normal cells recognized by immune cells) and upregulate immunogenic cell surface molecules such as MHC-I and II, introducing CAP in this field might lead to interesting outcomes. Other methods could include loading the inactivated whole-cell cancer stem cells with lipid nanoparticles containing anti-cancer agents or loading the cancer stem cells with liposomes containing iPSCs, followed by their injection; this could improve the efficiency of DCs and thus the immunogenic response. Moreover, this method may be able to target several factors at the same time; for example, both inactivated cancer stem cells and iPSCs cover a wide range of tumor antigens by themselves, which are poorly immunogenic (do not scape the cancer immune cycle) [185,187,424], and if they are used together in a system, this coverage spectrum might be increased and help to solve tumor heterogenicity problems. Furthermore, this combination system could further enhance the immunogenic response to prevent cancer recurrence. In the end, if the cold plasma is integrated with such a combination system, the combination system could be improved even more, as the CAP itself selectively destroys the tumor cells. At the same time, the liposomes containing iPSCs penetrate the DCs and enhance the variability of tumor antigen presentation, followed by their detection by T cells and the immune response. These are some of the methods that require future investigations, which might open novel and effective approaches to therapeutic cancer vaccine development.

## 8. Summary and Conclusions

To summarize, we have highlighted the recent research progress of four major vaccine platforms and their limitations. We believe that a comprehensive understanding of the immunosuppressive tumor microenvironment is essential for developing effective cancer vaccines. Besides, particle-based delivery systems have been intensively studied for cancer vaccines in the past few decades, and these hold great promise for improving the immunogenicity of vaccines and facilitating lymph node transport. There is already a consensus that cancer vaccines could achieve a greater therapeutic effect if they were administered in combination with other immunomodulation or standardized therapies. However, sustained endeavors are still needed for identifying tumor-specific neoantigens, effective adjuvants, and optimizing delivery platforms.

## Figures and Tables

**Table 1:** Comparing different vaccine platforms.

	Advantages	Disadvantages	Status of Some of the Vaccines in Clinical Trials (2016–2023)
Peptide vaccines	Simple chemical-based synthesis Cost-effectiveness Flexibility to multiple antigens High specificity High stability Safety for clinical applications Induce both humoral and adaptive immunity systems [106,405,406,407,408,409,410]	Relatively poor immunogenicity Inappropriate adjuvants Tumor heterogeneity Antigen loss Lower MHC expression Lack of T cell infiltration in the tumor tissue Inducing immune suppression through T cell dysfunction There is no FDA approved in vivo peptide-based cancer vaccines [106,411,412]	Glioblastoma/Glioma: Phase I (NCT05283109, NCT05283109, NCT04280848, NCT04116658, NCT04943718, NCT02960230) Phase II (NCT04280848, NCT04116658, NCT03018288, NCT02960230) Phase III (NCT03149003) Breast Cancer: Phase I (NCT05269381, NCT02938442), Phase II (NCT02938442, NCT03012100, NCT03606967, NCT02636582, NCT04197687, NCT03606967) Cervical/Uterus/Ovarian Cancers: Phase I (NCT05269381, NCT04580771, NCT03728881, NCT02865135, NCT03311334, NCT03761914, NCT02785250, NCT03206047), Phase II (NCT03728881, NCT04445064, NCT03946358, NCT02865135, NCT03311334, NCT03029403, NCT03761914, NCT02785250, NCT03206047, NCT04713514) Phase III (NCT04782895, NCT04508309) Lung Cancer: Phase I (NCT05269381, NCT02818426, NCT03715985), Phase II (NCT02818426, NCT04263051, NCT03715985) Phase III (NCT02654587, NCT04998474) Prostate Cancer: Phase I (NCT05010200) Phase II (NCT03579654, NCT04114825) Leukemia/Blood Cancer: Phase I (NCT03559413, NCT03761914), NCT05025488, NCT04688385) Phase II (NCT04747002, NCT03560752, NCT03559413, NCT03761914, NCT04060277, NCT03702231, NCT02802943) Head and Neck Cancer: Phase I (NCT02865135, NCT03821272, NCT05269381) Phase II (NCT03946358, NCT04369937, NCT02865135, NCT03821272, NCT04445064) Gastric Cancer: Phase I (NCT05269381) Bladder Cancer: Phase I (NCT05843448, NCT03715985, NCT05843448) Phase II (NCT03715985) Liver Cancer: Phase I (NCT05059821) Phase II (NCT04206254) Phase III (NCT04206254) Colorectal Cancer: Phase I (NCT03761914) Phase II (NCT03761914) Melanoma Cancer: Phase I (NCT05269381, NCT03715985) Phase II (NCT03715985)
Viral/bacterial-based vaccines	Recapitulate the natural infection process of specific pathogens Induce strong and long-lasting immune responses Produce high immunogenicity without adjuvant Flexible and facile engineering possibility for designing more selective vaccines [413,414,415]	Not a robust immune response in some cases Pre-existing immunity to the viral vectors Limited capacity for gene insertion Limited expression of viral transgene due to lysis of the target cell [363,416]	Pancreatic Cancer: Phase I (NCT03329248, NCT03136406, NCT03953235, NCT05076760), Phase II (NCT03329248, NCT03329248, NCT03953235) Glioblastoma/Glioma: Phase II (NCT04105374) Phase III (NCT04105374) Breast Cancer: Phase I (NCT05076760) Phase II (NCT03632941) Prostate Cancer: Phase I (NCT03815942, NCT02649855, NCT05553639, NCT02933255) Phase II (NCT03815942, NCT03315871, NCT02649855, NCT02933255, NCT05553639) Cervical/Uterus/Ovarian Cancers: Phase II (NCT03113487) Lung Cancer: Phase I (NCT03953235, NCT05076760) Phase II (NCT03953235) Head and Neck Cancer: Phase I (NCT05076760) Gastric Cancer: Phase II (NCT04111172) Colorectal Cancer: Phase I (NCT03563157) Phase II (NCT03563157) Melanoma Cancer: Phase I (NCT05076760, NCT04410874) Phase II (NCT04410874)
DNA vaccines	Cost-effectiveness Repetitive administration possibility Simple and flexible design Encoding different antigens Triggers long-lasting innate and adaptive immune responses Devoid of pathogenic infection or clinical side effects Heat stable Facile transportation and storage Large-scale production possibility [417,418,419,420]	Poor immunogenicity Requires a carrier for delivery Inefficient APC-mediated antigen uptake Inefficient immune responses Risk of integrating into the host’s chromosomal DNA and insertional mutagenesis Expression of antibiotic-resistant genes [287,418,419]	Pancreatic Cancer: Phase I (NCT03122106, NCT04853017, NCT05726864) Phase II (NCT05726864) Glioblastoma/Glioma: Phase I (NCT03491683, NCT04015700, NCT03750071, NCT05698199) Phase II (NCT03491683, NCT03750071) Breast Cancer: Phase I (NCT02780401, NCT03199040, NCT03199040) Phase II (NCT05455658, NCT04329065) Prostate Cancer: Phase I (NCT03532217, NCT04989946) Phase II (NCT03600350, NCT04090528, NCT04989946) Cervical/Uterus/Ovarian Cancers: Phase I (NCT03444376, NCT04131413, NCT04853017) Phase II (NCT03444376, NCT03439085, NCT03439085, NCT03911076, NCT03823131, NCT05334706, NCT03946358, NCT05799144, NCT03911076, NCT05334706) Phase III (NCT03721978) Lung Cancer: Phase I (NCT03166254, NCT05726864, NCT04853017) Phase II (NCT04397003, NCT05242965, NCT05726864) Head and Neck Cancer: Phase II (NCT03823131, NCT03946358, NCT05799144) Melanoma Cancer: Phase I (NCT03289962, NCT03655756, NCT04160065) Phase II (NCT03897881, NCT04526899, NCT04079166)
mRNA vaccines	Encoding and expressing TAA, TSA, and their related cytokines Stronger humoral and cellular immunities compared with the pathogen and peptide-based vaccines Rapid production Low manufacturing costs [324,421]	In vivo instability of mRNA Insufficient mRNA distribution Inducing unwanted immune responses Possibility of vascular blockage due to combination of mRNA with serum proteins [383,422,423].	Pancreatic Cancer: Phase I (NCT04161755, NCT03948763, NCT04741984) Glioblastoma/Glioma: Phase I (NCT05938387, NCT04573140, NCT04741984, NCT04911621) Phase II (NCT03927222, NCT03688178, NCT04911621) Breast Cancer: Phase I (NCT03788083) Prostate Cancer: Phase I (NCT04382898, NCT04382898) Phase II (NCT04382898, NCT04382898) Cervical/Uterus/Ovarian Cancers: Phase I (NCT04163094, NCT03323398, NCT04163094) Phase II (NCT03323398) Lung Cancer: Phase I (NCT03639714, NCT03164772, NCT03289962, NCT05660408, NCT03948763) Phase II (NCT03639714, NCT03164772, NCT05660408) Head and Neck Cancer: Phase II (NCT04534205) Colorectal Cancer: Phase I (NCT03948763, NCT03948763) Phase II (NCT04486378, NCT05456165)
Cell-based vaccines (DCs, iPSCs, and CAR-T cells)	Presenting all potential antigens to the immune system Producing various tumor antigens Long-lasting immune responses Mimicking the expression of tumor-cell antigens Inducing significant antitumor immune responses (in particular in iPSCs) [187,424]	Expensive Longer treatment duration (in case of iPSCs or CAR-T cell) Loss of the antigen recognized by CAR Cytokine-related toxicities (in case of CAR-T cell therapy) [201]	Pancreatic Cancer: Phase I (NCT02451982, NCT03767582, NCT03387098, NCT03552718) Phase II (NCT03190265, NCT02648282, NCT02451982, NCT03767582, NCT03161379, NCT03387098) Breast Cancer: Phase I (NCT03328026, NCT03552718, NCT03674827, NCT03387085, NCT05269381, NCT05035407) Phase II (NCT03328026, NCT03384914, NCT05455658, NCT03387085) Colorectal Cancer: Phase I (NCT03552718) Phase II (NCT04912765, NCT02919644) Head and Neck Cancer: Phase I (NCT03552718) Phase II (NCT04166006, NCT04445064) Glioblastoma/Glioma: Phase I (NCT04388033, NCT04388033, NCT04911621, NCT03914768, NCT04388033, NCT03914768) Phase II (NCT04523688, NCT04388033, NCT03395587, NCT03548571, NCT02465268, NCT03400917, NCT04388033, NCT04911621, NCT04388033) Phase III (NCT03548571) Lung Cancer: Phase I (NCT03674827, NCT05035407, NCT03970746, NCT04487756, NCT02466568, NCT03674827, NCT05104515, NCT05035407) Phase II (NCT03970746, NCT03406715, NCT04487756, NCT04277221, NCT02466568, NCT04998474, NCT04300244, NCT05242965) Liver Cancer: Phase I (NCT03552718, NCT03552718, NCT03674073, NCT05059821) Phase II (NCT04912765, NCT04317248, NCT03406715) Cervical/uterine/Ovarian Cancers: Phase I (NCT05269381, NCT05035407, NCT05104515, NCT05035407) Phase II (NCT04800978) Gastric Cancer: Phase I (NCT04567069, NCT05035407) Phase II (NCT04567069) Leukemia/Blood Cancer: Phase II (NCT03059485, NCT04977024) Melanoma Cancer: Phase I (NCT03552718, NCT05269381)
